# Ikk2 regulates cytokinesis during vertebrate development

**DOI:** 10.1038/s41598-017-06904-7

**Published:** 2017-08-14

**Authors:** Hongyuan Shen, Eun Myoung Shin, Serene Lee, Sinnakaruppan Mathavan, Hiromi Koh, Motomi Osato, Hyungwon Choi, Vinay Tergaonkar, Vladimir Korzh

**Affiliations:** 1 Institute of Molecular and Cell Biology, Singapore, Singapore; 2 Genome Institute of Singapore, Singapore, Singapore; 3 Saw Swee Hock School of Public Health, National University of Singapore, Singapore, Singapore; 4 International Institute of Molecular and Cell Biology, Warsaw, Poland; 5 Cancer Science Institute, NUS, Singapore, Singapore; 6 Department of Biochemistry, NUS, Singapore, Singapore; 7 Center for Cancer Biology, Unisa, Adelaide Australia

## Abstract

NFκB signaling has a pivotal role in regulation of development, innate immunity, and inflammation. Ikk2 is one of the two critical kinases that regulate the NFκB signaling pathway. While the role of Ikk2 in immunity, inflammation and oncogenesis has received attention, an understanding of the role of Ikk2 in vertebrate development has been compounded by the embryonic lethality seen in mice lacking Ikk2. We find that despite abnormal angiogenesis in IKK2 zygotic mutants of zebrafish, the maternal activity of Ikk2 supports embryogenesis and maturation of fertile animals and allows to study the role of IKK2 in development. Maternal-zygotic *ikk2* mutants represent the first vertebrates globally devoid of maternal and zygotic Ikk2 activity. They are defective in cell proliferation as evidenced by abnormal cytokinesis, nuclear enlargement and syncytialisation of a significant portion of blastoderm. We further document that reduced phosphorylation of Aurora A by Ikk2 could underlie the basis of these defects in cell division.

## Introduction

A number of cellular and extracellular signals trigger the activation of the NFκB transcription factors^1, 2^. The IκB kinase (IKK) complex consisting of core components such as IKK1 (IKBKA) and IKK2 (IKBKB) and non-catalytic components such as NEMO (IKBKG), Rap1^3^ and ELKS^4, 5^ plays a critical role in activation of NFkB. Signaling downstream of IKK2 is largely termed as the canonical NFκB signaling, whereas IKK1-mediated regulation of NFkB pathway is classified as the noncanonical NFκB signaling^6–9^. Kinase activity of IKK2 is known to be required for transcription of genes involved in innate immunity, inflammation, as well as development^6, 10–15^. While IκB proteins are the key targets of IKK2, other signaling proteins such as p53^16^, p65^3^ and Aurora A^17^ have been linked to key physiological processes downstream of IKK2. It has been documented that Ikk2 regulates cytokinesis by stabilizing Aurora A and Kif11 to maintain the bipolar spindle *in vitro* ^17^.

Although IKK2 loss-of-function (LOF) mutations in human patients have been documented^18–20^, effects of pan-embryonic deficiency of Ikk2 in vertebrates remains poorly characterized as severe liver apoptosis in mice precludes their analysis^6^. Hence the roles ascribed to IKK2 are largely extrapolated from *in vitro* experiments or tissue-specific mutagenesis^21, 22^. In particular, epidermis-specific deletion of Ikk2 causes severe inflammatory skin disease^23^, while deficiency of Ikk2 in endothelium results in vascular permeability and reduces cell migration traced to transcriptional regulation of HIF-1α^24, 25^. Despite these results, a thorough understanding of the developmental function of IKK2 in a global is lacking.

Zebrafish has been established as a useful vertebrate model to study a role of genes during development^26–28^. Recent introduction of efficient methods of gene-specific mutagenesis has made zebrafish particularly useful for analysis of mutations, which lead to embryonic lethality in mice since the early development of zebrafish is less dependent on circulation^29–31^. At midblastula transition (MBT) cells of early zebrafish embryo segregate into three different lineages – deep cells, which form the embryo proper, and two extraembryonic lineages – yolk syncytial layer (YSL) and enveloping layer (EVL)^32^. EVL forms an external layer of epidermis (periderm), which is thought to play an evolutionarily conserved role in communication with epidermis. Stabilization of both differentiating epidermis and epithelial–mesenchymal interaction with the TNF signaling plays a pivotal role in periderm homeostasis^33–37^. Microtubules are involved in the intracellular transfer of vesicle-packed membrane components towards the furrow and, thus, play an important role in blastomeres cohesion during first cell divisions. Microtubules deficiency causes the premature and excessive formation of the YSL and axis deficiency^38, 39^.

Previously, the inhibition of the Ikk2 action towards NFκB in zebrafish revealed its role in axis formation^40^. To analyze the NFκB-dependent and -independent roles of Ikk2 during early development in an unbiased manner, we used the zinc-finger nuclease (ZFN)-mediated mutagenesis to generate the two different mutant alleles of Ikk2. Their analysis demonstrates that the zygotic function of Ikk2 is required during embryogenesis to establish vascular integrity. Some of the zygotic Ikk2 mutants survive until sexual maturity. A significant portion of these animals develops skin lesions and deformed body axis. Further analysis of their progeny (MZ*ikk2* ^−/−^) totally devoid of Ikk2 activity reveals a role of Ikk2 in cytokinesis, epiboly (gastrulation) and axis development.

## Results and Discussion

**Figure 1:**
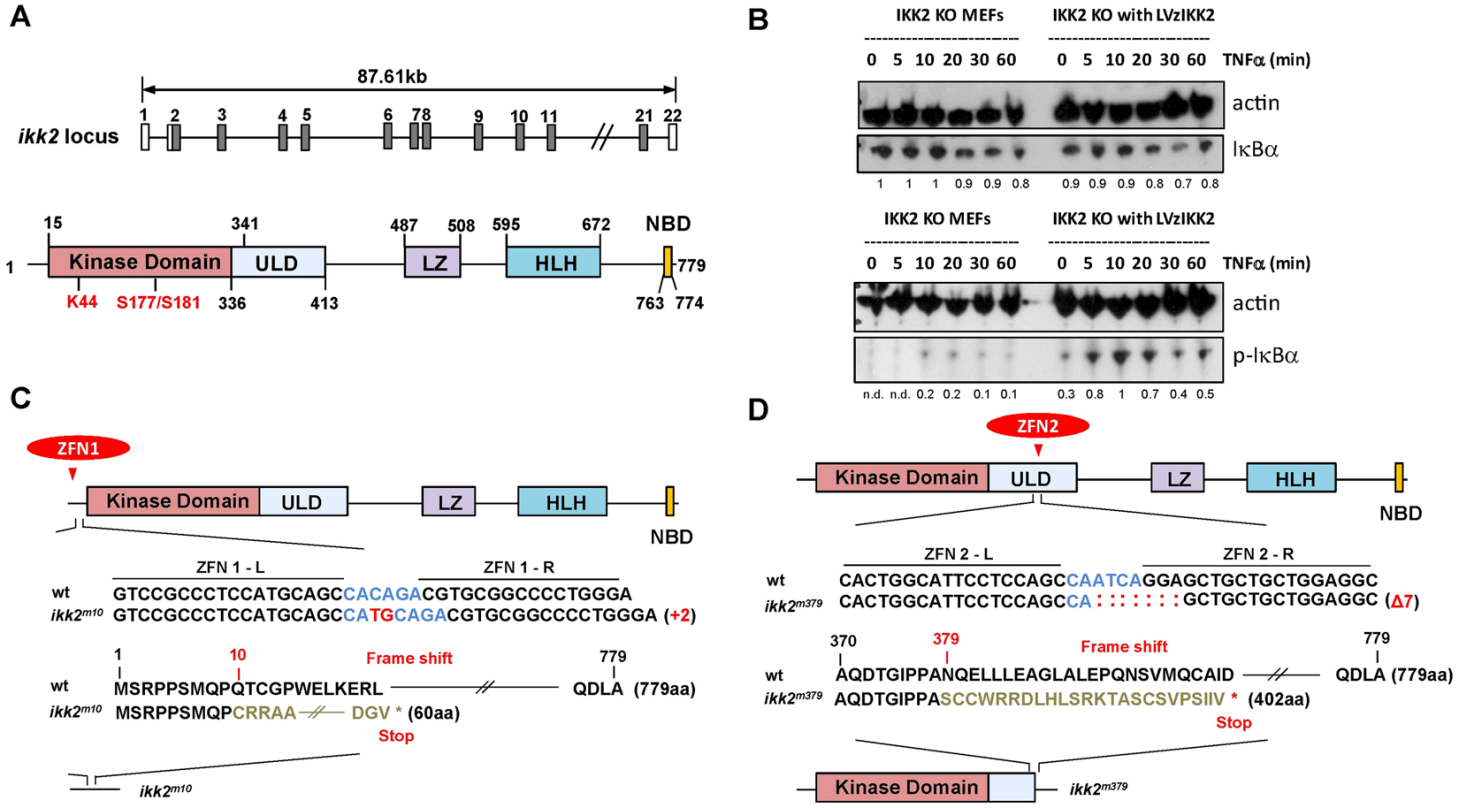
The two alleles of zebrafish *ikk2* (*ikbkb*) mutant made using zinc-finger nuclease (ZFN)-mediated mutagenesis. (**A**) Genomic organization of the zebrafish *ikk2* locus. It contains 22 exons (numbered). The domain organization of a putative Ikk2 of 779 aa derived by homology comparison with human IKK2. Numbers define amino acid residues. Abbreviations: ULD – ubiquitin-like domain; LZ - leucine zipper; HLH - helix-loop-helix domain; and NBD - NEMO-binding domain. The critical amino acid residues conserved in evolution are shown in red. (**B**) Zebrafish Ikk2 restored phosphorylation of murine IκBα in IKK2^−/−^ immortalized MEFs upon treatment with TNFα for the indicated time period. Actin was used as loading control. Band intensities were quantified by densitometry in ImageJ and normalized to the most intensive band for each target. n.d., not detected. Data are representative of three experiments. (**C**) The first ZFN-mutation upstream of the kinase domain (*ikk2* ^*m10*^) caused an indel mutant with a frame shift from 10 aa onwards and a truncated polypeptide of 60 aa. (**D**) The second ZFN-mutation designed within the ubiquitin-like domain (*ikk2* ^*m379*^) caused an indel mutant with a frame shift from 379 aa onwards and a truncated polypeptide of 402 aa.

To study the role of Ikk2 in development, we cloned it from total RNA of 96 hpf zebrafish. Zebrafish Ikk2 is homologous to the mammalian Ikk2 with 75% identity (Fig. S1A). The genomic locus of *ikk2* contains 22 exons spanning 87.61 kb. It encodes a polypeptide of 779 aa residues that contains evolutionarily conserved kinase, ubiquitine-like (ULD), leucine zipper (LZ), helix-loop-helix (HLH) and NEMO-binding (NBD) domains (Figs 1A; S1A). Notably, lysine 44 of the catalytic active site and the two serine residues (S177/S181) of the activation loop are conserved between zebrafish and other species Ikk2s. Lentivirus-mediated reconstitution of zebrafish Ikk2 in Ikk2-null murine fibroblasts led to IκBα phosphorylation and subsequent degradation upon induction with physiological stimuli TNFα, with kinetics similar to that seen in mammalian cells (Fig. 1B). This result suggested that the zebrafish Ikk2 is physiologically functional as a kinase and operates with similar efficiency as murine Ikk2 in activating the canonical NFκB signaling.

*ikk2* transcript is maternal suggesting its role during early development. It expresses throughout embryogenesis and in adults (Fig. S1B,C). To address a developmental role of Ikk2 and increase our chances to cause a developmental defect linked to Ikk2, we generated two mutant alleles using the zinc-finger nuclease (ZFN)-mediated mutagenesis^29, 30^. The first mutation was designed to be in the second exon, which corresponds to the N-terminal of the kinase domain. In this position, the 2-base pairs (bp) insertion typical of non-homologous end joining (NHEJ) repair was detected. This resulted in a shift of the reading frame causing an amino acid (aa) residue replacement (Q10C) and termination of a polypeptide after 60 aa (Figs 1C; S1D). This mutation (*ikk2* ^*m1*^ *°*) probably represents a null mutation (*sq302*). The second mutation was designed to be in the ULD. Analysis of this mutant allele demonstrated a 7 bp deletion, resulting in a frame shift, causing replacement of aa residue (N379S) that may terminate the polypeptide after 402 aa resulting in a truncated Ikk2 containing an intact kinase domain (*ikk2* ^*m379*^, *sq303*; Figs 1D; S1D), but without the important C terminal domain required for regulation of IKK2 activity^41^. When crossed to homozygosity, we observed no obvious difference in recessive mutant phenotypes displayed by the two mutant alleles and mutants generated by complementation crosses (*ikk2* ^*m10/m379*^). This is probably due to the fact that the loss of C-terminal domain in *ikk2* ^*m379*^renders the truncated kinase domain almost inactive. This is consistent with the extensively analyzed truncated human patient allele (Q432, premature stop) resulting in complete loss of IKK2 in fibroblasts and peripheral peripheral-blood mononuclear cells and impaired NFκB signaling downstream of TNFα/TLR5^18^. Therefore, unless otherwise stated, our analyses of zebrafish *ikk2* ^−/−^ is based on the putative null allele (*ikk2* ^*m10*^).

**Figure 2:**
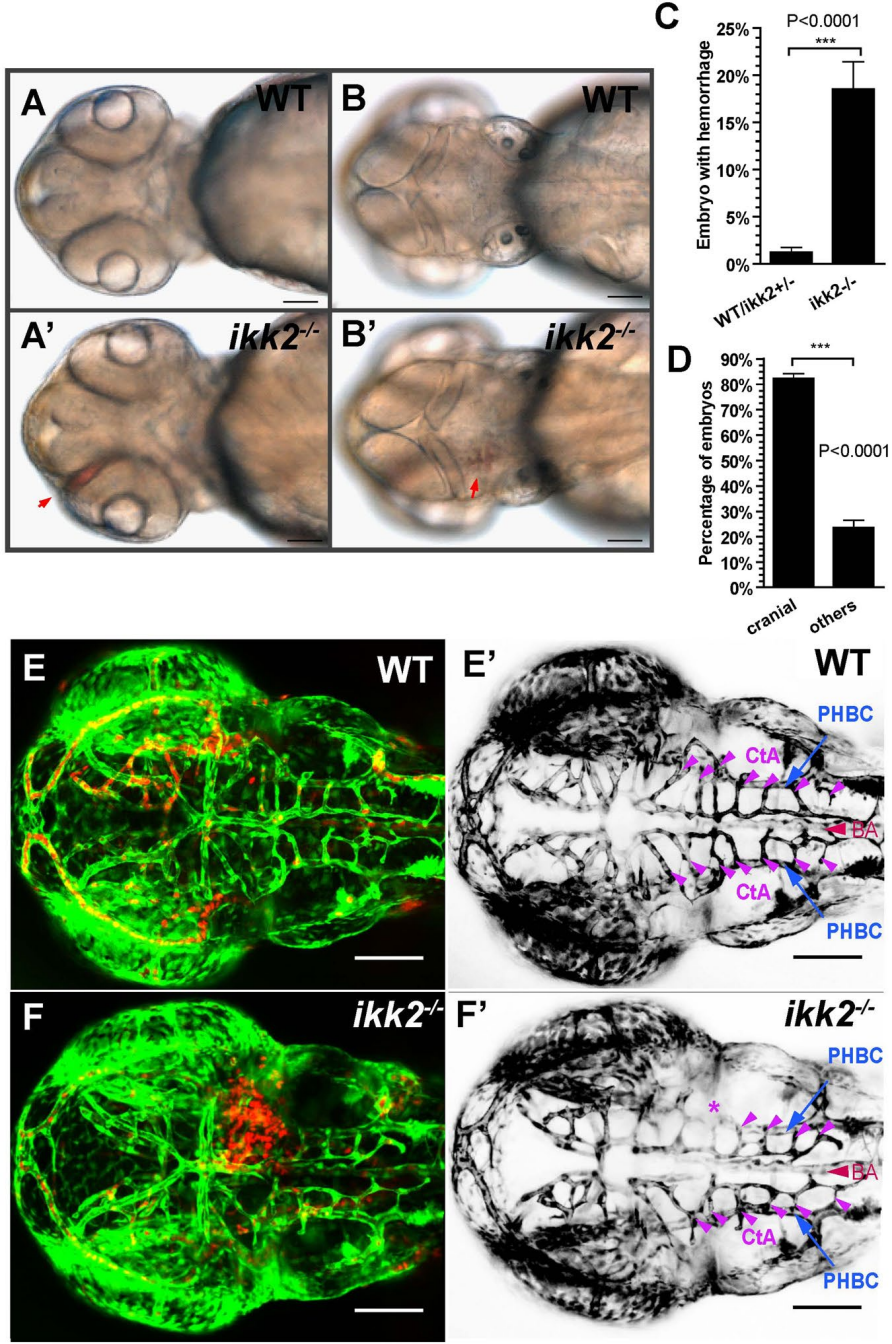
Loss of Ikk2 caused hemorrhage. (**A**,**B**) Bright-field images of wild-type and *ikk2* ^−/−^ (zygotic) embryos obtained from heterozygotic parents at 48 hpf. Red arrows - the hemorrhage site. (**A**,**A**′) Ventral view. (**B**,**B**′) dorsal view. (**C**) Quantification of hemorrhaging embryos. (**D**) Quantification of embryos with cranial hemorrhage vs that in other parts of the body. (**C**,**D**) Unpaired Student’s t test. (**E**,**F**) Confocal Z-projection shows wild type (**E**) and *ikk2* ^−/−^ (**F**) embryos of the Tg(fli:EGFP)/Tg(gata:DsRed) background at 48 hpf. (**E**′,**F**′) Confocal Z-projection shows Tg(fli:EGFP) embryos without the dorsal most MtA, MsA and DLV. EGFP was pseudo-colored as black. PHBC (blue), BA (red), and CtAs (purple) were marked. * - the affected CtAs with cranial hemorrhage in (**F**). All data expressed as mean ± SEM; scale bar in all panels: 100 μm. Abbreviations – PHBC: primordial hindbrain channels; BA: basilar artery; CtAs: central arteries (51).

**Figure 3:**
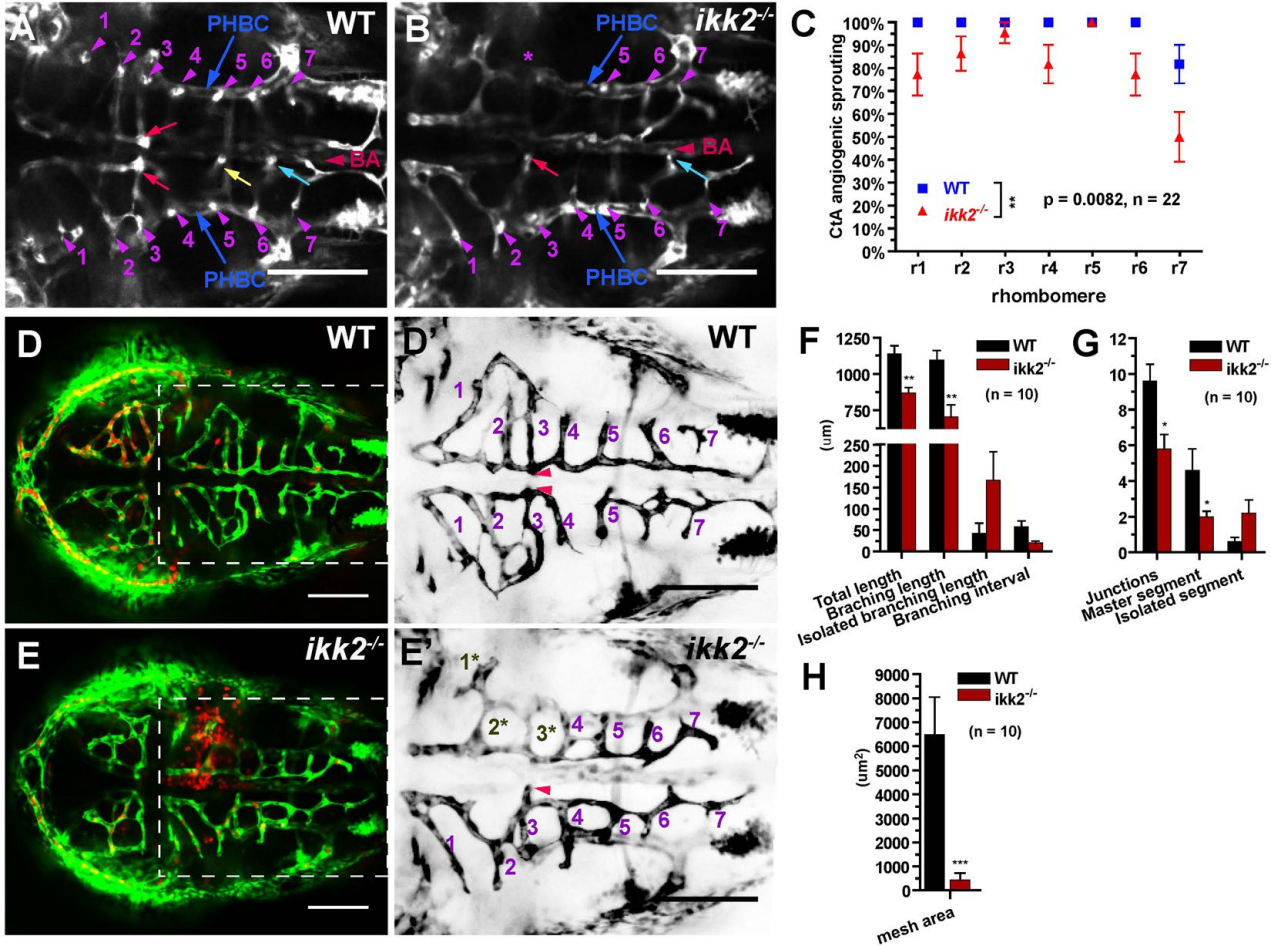
Loss of Ikk2 affects angiogenesis. (**A**,**B**) Confocal horizontal section of ventral hindbrain in wild-type and *ikk2* ^−/−^ (zygotic) embryos obtained from heterozygotic parents of Tg(fli:EGFP) background at 48 hpf. CtA (purple arrowhead) stem sprouting from PHBC in each rhombomere (r) was marked accordingly. At this stage, the bilateral CtAs in the r3 formed two connections with lumen to BA (yellow arrow) in wild-type and only one in *ikk2* ^−/−^ embryo. Connections of CtAs in r5 (yellow) and r6 (blue) marked. (**C**) Quantification of CtA sprouting from PHBC in each rhombomere. P value calculated by paired Student’s t test. (**D**,**E**) Confocal projection of CtA angiogenic vessels in Tg(fli:EGFP)/Tg(gata:DsRed) 48 hpf embryos. (**J**′,**K**′) Blowup shows the hindbrain of Tg(fli:EGFP)- labelled CtA vessels. Numbers correspond to the rhombomeres containing CtA sprouts. Note that 1*, 2*, 3* marked the CtA vessels that were reduced both in size and connectivity in *ikk2* ^−/−^ embryos. This is the area of hemorrhage (**E**). (**F**–**H**) Quantification of angiogenic index of hindbrain CtAs. P value calculated by Student’s t test. All data are expressed as mean ± SEM; scale bar in all panels: 100 μm. Abbreviations – PHBC: primordial hindbrain channels; BA: basilar artery; CtAs: central arteries (51).

A significant number of *ikk2* ^−/−^ embryos (18.5%) developed hemorrhage at 48 hpf, with 82.2% of cases being cranial (Fig. 2A–D). We crossed the *ikk2* ^*m10*^ mutant to *Tg(fli:EGFP)*/*Tg(gata:DsRed)* background, which labels endothelial cells and erythrocytes respectively, for detailed confocal analysis of vessel patterning in the hindbrain. The major arteries (BA) and veins (PHBC) were intact, hence vasculogenesis in the mutant was not affected (Fig. 2E–F). However, capillaries (CtAs) sprouting from existing veins (PHBC) were reduced in both number (Fig. 3A–C) and complexity (Fig. 3D–H; Fig. S2A–F). This implies that the hemorrhage observed in mutants could be due to reduced sprouting of capillaries and insufficient development of capillary network, a phenotype normally associated with angiogenesis defects. Previously, NFκB activation has been linked to maintaining blood vessel integrity in zebrafish through Birc2/TNF receptor complex^42^. In mice, deficiency of Ikk2 function in endothelium causes vascular permeability and reduces cell migration traced to regulation of HIF-1α^24, 25^. Cranial hemorrhages have also been described in human patients with IKK2 mutation^18^. Our observation of endothelial defects in zygotic Ikk2 mutants supports these observations and links this phenotype to several genes of angiogenesis (see below).

**Figure 4:**
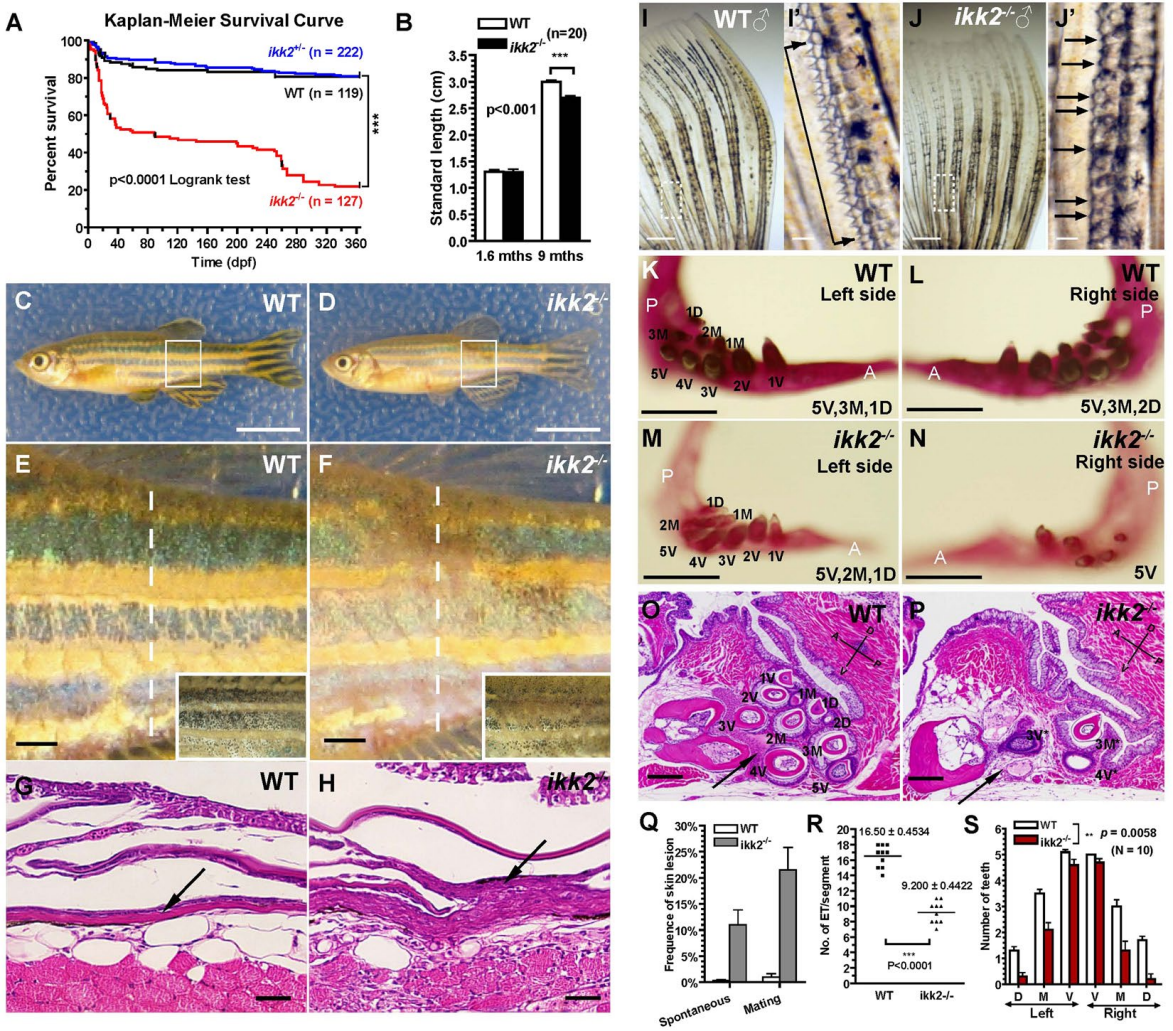
Ikk2 regulates development of skin and its appendages. (**A**) Kaplan-Meier analysis of survival of *ikk2* ^−/−^ fish during one-year period. P < 0.001, by Logrank test. (**B**) Nine months-old adult *ikk2* ^−/−^ (zygotic) mutants obtained from heterozygotic parents are small. P < 0.001, unpaired t test. (**C**–**F**) Adult *ikk2* ^−/−^ fish are hypopigmented (boxed area and inset, E,F). They succumb to skin injuries without or with mating, with an average frequency of 11% and 21.5%, respectively. Scale bar in (**C**,**D)**: 1 cm; in (**E**,**F)**: 1000 µm. (**Q**). (**E**,**F**) blowup of white rectangle area in (**C**,**D)**. In severe cases, the injuries cause skin lesion. (**G**,**H**) H&E stained cross-section at the level of skin lesion area (dashed line in **C**,**D**). Scale bar: 500 µm. (**I**,**J**) Epidermal tubercles (ET) were reduced in *ikk2* ^−/−^. (**I**′,**J**′) Blowup of white rectangle area in (**I,J**). Quantifications by unpaired t test were shown in (**R**) Scale bar in I,J: 500 µm; in (**I**′,**J**′): 100 µm. (**K**–**N**) Alizarin red staining of teeth. Scale bar: 500 µm. (**O**,**P**) H&E stained sagittal section at the Vth branchial arch. Teeth numbers were marked by *. Note that in *ikk2* ^−/−^ fish, 4 V* was absent, 3 V* was defective, and blood supply to the dentation area (arrow) compromised. Scale bar: 100 µm. (**S**) Teeth number was significantly reduced in adult mutants. P = 0.0058, paired t test.

A small percentage of *ikk2* ^−/−^ embryos (~5%) showed phenotype similar to that of the *ntl* mutants, which have truncated body axis due to deficiency of the notochord^43^, with various degree of penetrance (Fig. S3A–H). They develop loss of cells during late epiboly, abnormal Kuppfer’s vesicle, *ntl* phenotype and vacuolated notochord (Fig. S3A–H and not shown). Similar phenotype was detected using the dominant-negative approach^9^ and transient morpholino-mediated knockdown of Ikk2 (Table S1). Hence it seems that the *ntl*-like phenotype develops due to incomplete inhibition of Ikk2-NFκB activity. In both embryos and adult mutants neither hepatic apoptosis nor liver failure were observed in *ikk2* ^−/−^ zebrafish. This finding is in accordance with IKK2-deficient human patients where no liver damage is reported^18–20^. It further confirms that unlike loss of NEMO or p65 lack-of-function that of IKK2 function could be tolerated. Embryonic apoptosis of hepatocytes resulting in lethality in Ikk2 KO mice could simply be due to excessive sensitivity of hepatocytes to TNFα during embryogenesis in mice. However, a significant reduction in mutant fish population was observed and the one year survival rate calculated to be mere 21.7% (Fig. 4A). Adult *ikk2* ^−/−^ fish were short, hypo-pigmented and prone to skin lesions (Figs 4B–F; S4A–F). Frequency of skin lesions increased during mating (Fig. 4Q), probably due to mechanical stress of copulation. Skin lesions in a significant number of fishes fail to heal and they die after a couple of weeks post injury. Injury sites showed pathological changes, including loss of scales, reduced skin parenchyma and hyper-proliferation of keratinocytes (Figs 4E–H; S4E,F). Lateral body muscles of the mutant fish showed less myofibrils and signs of inflammation with infiltration of blood cells (Fig. 4G,H). In addition, mutant males developed deformed and reduced in number breeding tubercles (keratinized cuticles present on dorsal pectoral fin of male zebrafish for grasping and positioning of females during mating, Fig. 4I,J). The pharyngeal teeth of mutants were also reduced in number (Fig. 4K–P,S). These signs indicated systemic deficiency of integument derivatives, including skin and skin appendages. This is consistent with published analyses of Ikk2-deficiency in mammals associated with severe inflammatory skin disease^18–20, 23^. This function seems to be different from that of Ikk1, which in zebrafish and mice controls epidermal differentiation independent of NFκB activation^6–8, 44^. Interestingly, in all IKK2 human patients analyzed so far, no anhidrotic ectodermal dysplasia (EDA) was reported^18–20^, suggesting a dispensable role of IKK2 for development of skin appendages. The skin phenotype of Ikk2 zebrafish mutant together with that in conditional KO mice^23^ probably implicates a more precise level of Ikk2/NFκB activity required for skin development in animals, which could somehow be tolerated in human. It is also possible that some of these skin defects could be due to defects in immunodeficiency (manuscript in preparation) or a combination of both.

**Figure 5:**
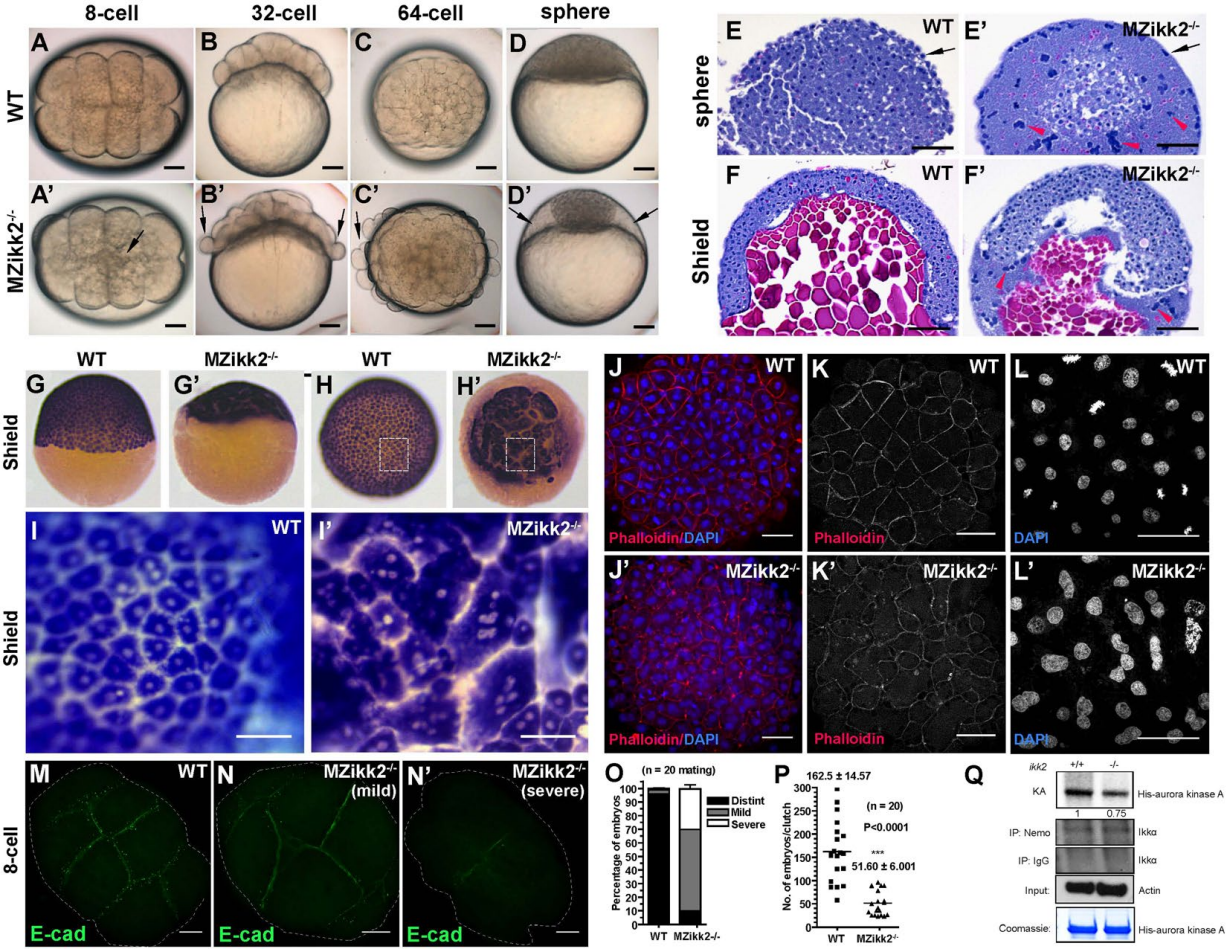
Cell adhesion and cytokinesis are affected in *MZikk2* ^−/−^ embryos. (**A**–**D**) Early cell adhesion and cytokinesis defects in *MZikk2* ^−/−^ embryos (obtained from homozygotic parents, arrow) were followed by the ‘cellular island’ phenotype (arrow) at sphere stage. (**E**,**F**) (**H**,**E)** stained sections showing abnormal nuclear clumps in the EVL (arrow) and enlarged YSL (arrowhead) of *MZikk2* ^−/−^ embryos. (**G**-**I**) *krt8* WISH reveals multinuclear cells in *MZikk2* ^−/−^ embryos. (**I**,**I**′) Blowup of boxed areas in (**H**,**H**′), respectively. (**J–L**) Whole-mount immunostaining using Phalloidin and DAPI shows cell boundaries (**K**,**K**′) and nuclei (**L**,**L**′) in *MZikk2* ^−/−^ embryos (animal pole view, sphere stage). (**M**,**N**) Cell division furrows stained by E-cadherin, 8-cell stage. (**O**) Quantification of embryos with mild and severe division furrow defects based on E-cadherin staining. (**P**) A number of embryos in *ikk2* ^−/−^ crosses as compared to wild-type; p < 0.001, Student’s t test. (**Q**) Ikk2-dependent phosphorylation of Aurora-A in extracts of *ikk2* ^−/−^ and controls. Actin and Coomassie blue, loading control. All data are expressed as mean ± SEM; Scale bar in all panels: 100 μm.

Ikk2-deficient adults could generate maternal-zygotic embryos (MZ*ikk2* ^−/−^) which allow us to study the global function of Ikk2 in the context of null background. When crossed, the *ikk2* ^−/−^ adults produce progenies with clutch size significantly smaller than controls (Fig. 5P), suggesting a functional role of Ikk2 in oogenesis. Indeed, real-time PCR (qPCR) indicated presence of maternal transcripts at as early as 1-cell stage (Fig. S6A). In such 8-cell stage embryos the cell boundaries were blurred (Fig. 5A′) and at the 32-64-cell stage cells were found to detach from each other (Fig. 5B′,C′). At the sphere stage, the mutant yolk syncytial layer (YSL) was abnormally large and cells formed a “cellular island” (Fig. 5D′), a characteristic phenotype of embryos with cell division defects^45^. By the time when the stage-matched controls complete epiboly, some of the MZ*ikk2* ^−/−^ embryos reached 50% epiboly and developed a ectopic yolk constriction that broke the yolk cell and caused early death (Fig. S5A–H). In summary, these phenotypes are observed consistently in crosses from different batches and usually 100% of MZ*ikk2* ^−/−^ embryos are affected. Occasionally (in one out of 10 crosses), a minority of MZ*ikk2* ^−/−^ embryos (less than 5%) develop past epiboly and develop the embryonic lethal *ntl-*like phenotype indicative of gastrulation deficiency (ref. 43; Fig. S4J–N; Table S1). Hence it is possible that this phenotype could also be due to incomplete inhibition of both maternal and zygotic Ikk2 activities.

Using whole-mount *in situ* hybridization (WISH) analysis we found relatively normal distribution of transcripts of a number of dorsal-ventral (*flh*, *chd and bmp2b*) and mesodermal markers (*ntl*, *papc*, *eve1*) in MZ*ikk2* ^−/−^ embryos at shield stage, suggesting that early embryonic patterning and differentiation still took place in the MZ*ikk2* ^−/−^ mutants (Fig. S5I–T). Interestingly, epidermal marker, *krt8*, which expression was absent in *ikk1* mutant^44^ was expressed (Fig. 5G,H). This provides evidence of keratinocytes differentiation in MZikk2^−/−^ embryos. As Ikk1 controls epidermal differentiation independent of canonical NFκB activation^7, 44^, the phenotype observed here could be attributed to the lack of Ikk2 function towards NFkB or other substrates, that the presence of Ikk1 catalytic activity cannot compensate for. Importantly, although the enveloping layer (EVL, primitive epidermis) was present, its cells were multinuclear (Fig. 5I,I′). Immunostaining with phalloidin and DAPI further revealed blurred cell boundaries and fused nuclei in the EVL (Fig. 5J–L). Sections of the sphere and shield stage MZ*ikk2* ^−/−^ embryos detected cytokinesis defects in deep cells also. The acellular area corresponding to YSL increased dramatically resulting in formation of the ‘cellular island’ phenotype (Fig. 5E,F). Taken together, these results suggest that cytokinesis defects could be the underlying primary defect in MZ*ikk2* ^−/−^ embryos.

Anti-Ecad immunohistochemistry revealed two distinct Ecad-positive subcellular signals in early WT embryos: a) the cell division furrow and, b) multiple small vesicles (with diameter below 10 μm) in close proximity to the furrow (Fig. 5M). In the MZ*ikk2* ^−/−^ mild mutant the small Ecad-positive vesicles were significantly reduced (Fig. 5N) and in the severe mutants the signals associated with cell division furrow was greatly reduced (Fig. 5N′,O). Formation of the cell division furrow depends upon vesicular transport and its irregularities were linked to deficient cytokinesis and enlarged YSL^38^.

Thus, the deficiency of cytokinesis led to expansion of the YSL. Although the molecular mechanism underlying cytokinesis has been a matter of intense investigation, the one underlying YSL formation has just begun to be elucidated^32, 46^. In embryonic zebrafish, the YSL forms due to regression of preexistent marginal cells membranes and depends on coordinated activity of different elements of the cytoskeleton. It was demonstrated that a disturbance of the cytoskeleton in embryonic zebrafish results in conversion of the blastoderm into syncythium. In particular, deficiency of several maternal proteins, including, centriolar protein Sas-6, Aurora-B kinase, and components of Rho signaling (Rho-kinase 1 and Slc3a2) was linked with excessive syncitialization of blastoderm^45, 47–49^.

The phenotype of MZ*ikk2* ^−/−^ embryos is strikingly similar to that of the MZ mutant of Aurora B^45^. It has been shown previously that a related protein Aurora-A plays a role in maintaining microtubule-based cytoskeleton during cell division and its inhibition decreases cell proliferation by inducing cell cycle arrest, polyploidy and affects the NFκB pathway^50^. To analyze a role of Ikk2 in zebrafish *in vivo*, Ikk complex was precipitated from adult *ikk2* ^−/−^ fish using anti-NEMO antibodies and Ikk activity was determined in an kinase assay using the recombinant full-length His-Aurora-A as substrate. Indeed kinase activity towards Aurora-A was reduced by 25% in the Ikk2 mutants suggesting subnormal functioning of the Ikk2-Aurora-A axis (Fig. 5Q). Deficiency of cytokinesis could be caused by abnormal microtubule-based cytoskeleton in MZ*ikk2* ^−/−^ mutants resulting in the “mitotic catastrophe” and termination of development.

**Figure 6:**
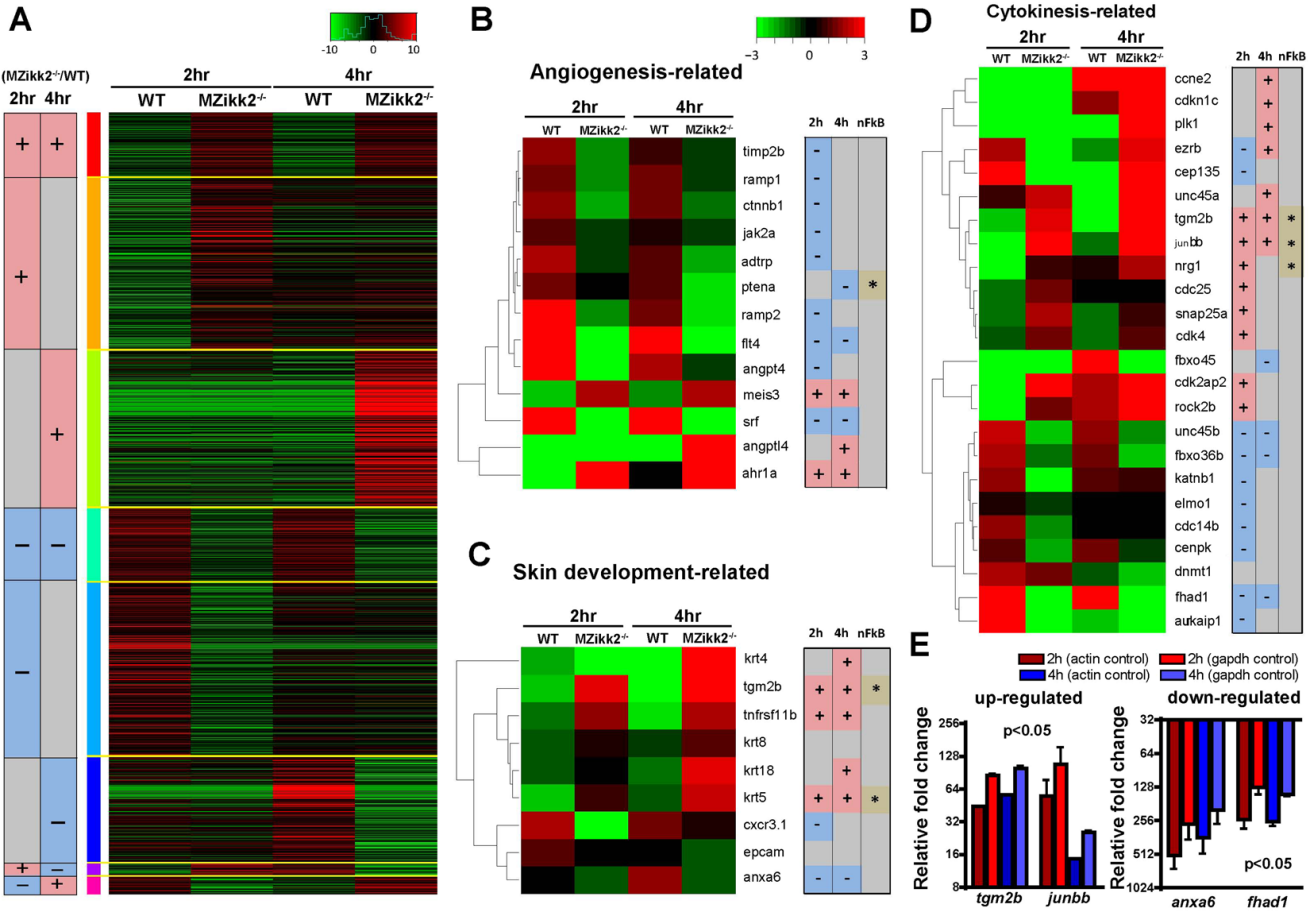
RNAseq analysis of transcriptome of wild-type and *MZikk2* ^−/−^ embryos (obtained from homozygotic parents) reveals novel molecular targets regulated by Ikk2. (**A**–**D**) Green - down regulated in *ikk2* ^−/−^ as compared to wild type control, red - up regulated in *ikk2* ^−/−^ as compared to wild type control. (**A**) Heat maps illustrating total stage- and gene-specific expression trends. (**B**) Comparison of expression of angiogenesis-related genes. (**C**) Comparison of expression of skin-related genes. (**D**) Comparison of expression of cytokinesis-related genes. (**E**) Real-time PCR (qPCR) verification of selected target genes identified by RNAseq. Two internal reference, *actb* (actin) and *gapdh* were used for normalization. Data quantification was done by using the ΔΔCt method and the result was presented as a relative fold change compared to wild-type embryos at 2 and 4 hpf. Data are expressed as mean ± SEM, p < 0.05 for all four genes listed.

Since MZ*ikk2* ^−/−^ embryos lack global Ikk2 activity, it opened a possibility to use RNAseq and identify the genes regulated by Ikk2 during oogenesis and early embryogenesis. We performed this analysis on the MZ *ikk2* ^*m10*^ mutants and controls at 2 hpf (prior to MBT, genes transcribed during oogenesis) and 4 hpf (post-MBT, same plus early embryonic genes; Fig. 6). Our analyses revealed that expression of genes related to angiogenesis (*ptena*, *angpt4*, *vegfaa*, *flt4*; Fig. 6B), skin development (*anxa6, tgm2b*, Fig. 6C), and cytokinesis (*junbb, etc*; Fig. 6D) were significantly affected. Expression of some of these genes was further validated by qPCR analyses (Fig. 6E). Some genes in these groups are known NFκB targets (*ptena, tgm2b, junbb)*. In addition, this analysis revealed a number of other genes/transcripts, which were not known previously as NFκB targets and yet their expression was significantly affected by the Ikk2 mutation (*fhad1*). Interestingly, the expression of three transcripts of *aurka* encoding the zebrafish homologue of Aurora kinase A (Aurora-A) changed in the MZ*ikk2* mutants along with expression of the negative regulator of *aurka* - *aurkaip1* (Fig. S6B). This suggested that regulation of Aurora-A kinase by Ikk2 probably takes place not only at the level of posttranslational regulation of Aurora-A (Fig. 5Q), but also at the level of transcription of *aurka* and *aurkaip1*. Amongst other regulators of mitosis Cdk4 plays a role in G-S1 phase^51^. Expression of *cdk4* was up-regulated significantly in MZ mutant embryos (Fig. 6D; Supplemental Table 1). It has been shown that early genes regulated by the NFκB-dependent TNFα-signaling in epidermal keratinocytes include BMP2^52^. This is consistent with the RNAseq data in MZ*ikk2* showing an up-regulation of *bmp2b* (GEO submission no. GSE90971). These preliminary observations defined tentative points of interest for future studies of the cytokinesis defect caused by a global deficiency of Ikk2 *in vivo* in zebrafish embryos.

A complex signalling pathway that controls many aspects of cellular functions and molecular interactions, NFκB pathway and its key molecular inter-players had been the focus of intensive studies over the last decades. Among them, the IKK complex which directly phosphorylate IκBα and allow NFκB protein translocation into nucleus has been one of them. Evidence from animal genetics (mainly mice) and cell-based biochemical studies point at IKK2 as the main catalytic active kinase, whereas IKK1 has some level of kinase activity with its own independent function and IKKγ functions as an essential regulator. Recent advance in human genetics leads to the identification human patients with IKK2 mutations^18–20^ and studies based on their phenotype seems to reinforce its role in immunity as patients all develop Severe Combined Immunodeficiency (SCID) within the first 6 month of age, which is attributed to the loss of function of canonical NFκB signalling. These data supports our previous understanding of IKK2 function gained from mice studies, but also emphasizes certain discrepancies. For example, none of patients display any hepatic problems whereas Ikk2 knockout mice is embryonically lethal due to TNFα-induced severe liver apoptosis, hence the prenatal function cannot be addressed. Although conditional knockout mice with immunodeficiency has been described^21, 22^, the exact molecular mechanism and pathological consequence due to loss of IKK2 at a systemic level remain to be elucidated. Our analysis of zebrafish Ikk2 mutant fits nicely into this gap. Zebrafish i*kk2* mutants develop haemorrhage at early embryonic stage and later succumb to systemic infection (our unpublished data) hence poor survivability, a phenotype very similar to that described in human patients. With large fecundity (~150–250 embryos per clutch) and ease of maintenance, zebrafish *ikk2* mutants represent an attractive vertebrate model for drug screening to be carried at at Ikk2/NFκB deficient background.

In *Drosophila*, the immune deficient (IMD) pathway (canonical NFκB pathway equivalent), is triggered by Gram-negative bacteria and activates Relish, a p100-like NFκB precursor protein, resulting in induction of antimicrobial peptide genes. The *Drosophila* Ikk complex contains 2 subunits, a catalytic kinase subunit encoded by *ird5* (IKK2)^53^ and a regulatory subunit encoded by *kenney* (IKKγ)^54^. The lack of IKK1 homologue in *Drosophila* reiterates the evolutionary conserved function of IKK2 as the key kinase that activates canonical NFκB signalling, yet calls for a more complex system with IKK1 functional interplay. Our zebrafish analysis fits nicely here. Our studies of MZ*ikk2* ^−/−^ mutants at a completely null background reveals a novel function of Ikk2 in regulating cytokinesis early in development. As zebrafish *ikk1* ^−/−^ mutants survive to adulthood without obvious morphological problem, whereas MZ*ikk1* ^−/−^ cannot differentiate into a proper embryonic ectoderm^44^, it is therefore obvious that Ikk1 and Ikk2 have their own individual roles in epidermal development, which possibly explains the skin phenotype observed at later stages in zygotic mutants as well as other in other model systems. Hence, the availability of both *ikk1* and *ikk2* maternal zygotic mutants in zebrafish offers us an unique peek into the function of these two kinases in a very clean background that other model systems do not offer. Taken together, the establishment of zebrafish *ikk2* mutants and their functional analysis offer an attractive alternative approach in understanding the physiological function of IKK2 that complements current knowledge based on mammalian models and changes our perspective on a developmental role of the NFκB signalling pathway in vertebrates.

## Material and Methods

### Zebrafish husbandry and transgenic lines

Wild-type zebrafish (AB strain) were maintained as described previously^55^. All animal experiments were carried according to the regulations of Institutional Animal Care and Use Committee (Biological Resource Center of Biopolis, license no. 120787), which approved this study. Developmental stages are in hours post fertilization (hpf) at 28.5 °C. Transgenic lines: *Tg(fli:EGFP)* and *Tg(gata:DsRed)* that label zebrafish vasculature and erythrocytes, respectively, were used for analyzing angiogenesis defects.

### Generation of zebrafish *ikk2* mutants using zinc-finger nuclease (ZFN)

#### ZFN design, assembly and validation

The two ZFN pairs used in this study were purchase from CompoZr Custom Zinc finger nuclease (Sigma-Aldrich, St. Louis, USA), with design, assembly and validation performed by the manufacturer. ZFN activities were measured using yeast MEL-I reporter assay by the manufacturer and were reported to be 68.3% and 118.2% relative to positive control for ZFN pair 1 (ZFN1, Fig. 1C) and ZFN pair 2 (ZFN2, Fig. 1D) respectively. Experiments were performed according to manufacturer’s guidelines.

#### Preparation and microinjection of ZFN mRNA

All pZFN plasmids were linearized with XbaI, for synthesis of ZFN encoding mRNA using mMessenger kit (Ambion) according to manufacturer’s protocols. Synthesized mRNAs were Poly (A) tailed with Poly (A) tailing kit (Ambion) and purified by phenol/Chloroform extraction. Paired ZFN mRNAs were mixed at a 1:1 ratio and 400 pg of RNA was injected into 1-cell staged zebrafish embryos. Embryos that survived into adulthood were screened for mutation in the respective ZFN target site.

#### ZFN induced mutation detection using SURVERYOR nuclease

The Surveyor Mutation Detection Kit (Transgenomic) was used to detect ZFN-induced mutation within *ikk2* exon 2. PCR reactions (50 µl) were carried out using DreamTaq polymerase (Fermentas) in the manufacturer-supplied buffer. Primer pairs flanking the *ikk2* exon 2 ZFN 1 target site (5′-GGGTAAATACAATTCAGAGCATG-3′, 5′-CTTTTACTGCTGAACTACACC T-3′) and ikk2 exon 11 ZFN 2 target site (5′-GCCATAAGTCACATGTCATGTT-3′, 5′-GTT AAAGCAACAGCTAGAGAGG-3′) were used. PCR reaction program: denature at 95 °C for 2 min followed by 35 cycles consisting of 95 for 40 s, 55 for 30 s and 72 for 30 s; 72 for 10 min final extension. PCR product was hybridized in a thermocycler following the protocol in the Surveyor manual. The hybridized DNA was assayed for inhomogeneity by adding 1 µl Surveyor Enhancer S, 1 µl Surveyor nucleases S and 2 µl of 0.15 M MgCl2 per 20 µl of DNA and incubating the reaction at 42 °C for 1hr. After incubation, treated DNA was assayed by 2% agarose TBE gel electrophoresis. Identified founders were out-crossed with wild-type AB fish to establish F_1_ generations. Exact indel mutant sequences were confirmed by cloning and sequencing. F_1_ generations were then subsequently out-crossed to wild-type AB strain for at least four generations for clearance of potential off-target background until linked mutant phenotype were confirmed. All genotyping were performed by sequencing (Fig. S1B).

### Cell lines, antibodies and other reagents

Wild type (WT) and *Ikk2* ^−/−^ mouse embryonic fibroblast (MEFs) were grown in DMEM supplemented with 10% heat inactivated fetal bovine serum, 2 mM L-glutamine, 2 mM Na pyruvate and 1x PSF (penicillin, streptomycin and fungizone) at 37 °C with 5% CO_2_. Antibodies against IKKα (Sc7182, Sc7218), IKKβ (Sc7607), IKKγ (Sc8330) were from Santa Cruz Biotechnology and Biosource. The anti-IKKα/β rabbit IgG antibody (sc-7607, Santa Cruz Biotechnology) recognizes human IKK1 and IKK2 both. IKKγ specific antibody was obtained from Biosource and Millipore. TNFα (Calbiochem) were used at concentration of 10 ng/ml.

### Lentivirus Production and Transduction

Production and purification of lentiviral vectors has been described previously. Briefly, lentiviral vector encoding the zebra fish Ikk2 and other control molecules and packaging plasmids (µg) were co-transfected into 293 T cells using calcium phosphate method. Culture media was changed after 12 hours and harvested at 24 and 48 hours before centrifuging to pellet the release virus. Viruses were re-suspended in 1.6 ml of 1x HBSS and aliquoted before freezing at 80 °C. Cells in 24-well plate was transduced with the lentivirus (50–100 µl/well) for 24 hours before changing media.

### Western Blot Analysis

Total protein was extracted with TOTEX buffer (20 mM Hepes pH 7.9, 0.35 M NaCl, 20% glycerol, 1% NP-40, 1 mM MgCl_2_, 0.5 mM EDTA, 0.1 mM EGTA, 50 mM NaF and 0.3 mM NaVO_3_) containing a mixture of protease inhibitors (Roche). 20 µg of total protein were separated on NuPAGE Novex 4–12% Bis-Tris gels (Invitrogen) and transferred to polyvinylidene difluoride membrane (Biorad). Immunoblotting were performed with specific antibodies and visualized using ECL Western Blotting detection kit (Amersham Bioscience).

### Co-Immunoprecipitation

At a desired point after infection/transfection, cells were washed with ice-cold PBS, and lysed in 10 mM Tris pH8, 170 mM NaCl, 0.5% NP40 and protease inhibitors for 30 minutes on ice. Cell lysates were cleared by centrifugation and the supernatants were incubated with anti-p65 antibody overnight at 4 °C and with protein-G sepharose for a further 2 hour. Beads were washed 4x with 1 ml of wash buffer (200 mM Tris pH 8.0, 100 mM NaCl and 0.5% NP-40). Bound proteins were eluted with SDS sample buffer and separated on NuPAGE Novex 4–12% Bis-Tris gels before immunoblotting with specific antibodies.

### Quantitative real-time PCR

Total RNA were isolated using RNeasy Kit (Qiagen) as per manufacturer’s protocol. cDNA were prepared from 1–2 µg of RNA using the Superscript III or superscript Vilo reverse transcriptase (Invitrogen) with random hexamers primers. Real time PCR reactions were performed in duplicates using SYBR Green (Biorad) or SYBR GreenER (Invitrogen) as per manufacturer’s instructions. Cycles for BioRad 60–95 °C for 30 sec followed by 40 cycles of 95 °C 15 Sec and 60 °C for 30 seconds. Cycles for SYBR GreenER: 7.5 mins at 95 °C for the initial denaturation, followed by 40 cycles of 95 °C for 15 seconds and 60 °C for 30 seconds. Primer sequences are available upon request^56^.

### *In-vitro* Kinase Assay

Total lysates prepared from female WT and *ikk2* ^−/−^ tissue were immunoprecipitated with anti-IKKγ/NEMO (sc-8330, FL-419, Santa Cruz) overnight at 4 °C. After extensive washing of the beads, kinase activity was determined by incubating beads with 1 μml^−1^ (^32^P) ATP and 2 μg of purified recombinant His-aurora kinase A (plasmid kindly provided from Edward Manser’s lab, IMCB, Singapore) for 30 minutes at 30 °C. Reactions were resolved on 15% PAGE gel and visualized by autoradiography as previously described^57^.

### Protein Expression and Purification

Proteins were expressed in BL21 cells grown at 37 °C to an OD600 = 0.6, then induced with 0.5 mM IPTG and incubated for a further 9 hours at 20 °C as previously described^57^. The GST-fusion proteins were purified on Glutathione Sepharose resin (Amersham Bioscience). The resin was washed with buffer A (50 mM Tris pH7.5, 1 mM EGTA, 1 mM EDTA and 2 mM DTT) followed by buffer B (50 mM Tris pH7.5, 200 mM NaCl and 2 mM DTT). Bound protein was eluted with elution buffer (50 mM Tris pH8.5, 10 mM glutathione). Eluted protein was filtered, concentrated and frozen in aliquots before use.

### Whole mount *in situ* hybridization (WISH), histology and immunohistochemistry

WISH, histology and immunohistochemistry were performed following established protocols. Briefly, for WISH, embryos were fixed in 4% paraformaldehyde in PBS for 4 hours at room temperature, and hybridized with specific probes in hybridization buffer (50% formamide, 5X SSC, 50 mg/ml Heparin, 500 μg/ml tRNA and 0.1% Tween20 in PBS) at 68 C. Stain was developed with NBT/BCIP (3 μl/ml of 50 mg/ml stock). All washings were done in PBS with 0.1% Tween20. Specifically, embryos at blastula and gastrula stages were fixed within their chorion on, whereas embryos after 24 hpf were de-chorionated and fixed. Anti-E-cadherin (mouse, #610181, BD transduction laboratories) primary antibodies were used for immunohistochemistry (1:200 in PBS), followed by secondary antibodies with AlexaFluor-488 (Invitrogen) (1:500 in PBS). AlexaFluor-555-conjugated Phalloidin and DAPI were used 1:500 in PBS. Confocal images were taken with Zeiss LSM710 confocal systems and processed using ImageJ.

### Skin and skin appendages analysis

To quantify ET units per segment, one pectoral fin from each animal was collected and regions of four segments proximal to the bifurcation point of the third and fourth fin rays were assessed. To visualize adult teeth, anesthetized fish were fixed in 4% PFA overnight and transferred into 0.01% alizarin red in 0.5% KOH for staining at 4  °C overnight. Following rinsing in 0.5% KOH, teeth from both side were removed from the fifth branchial arches and transferred into 50% glycerol for imaging.

### Survival curve, standard length and statistical analysis

Survival of individual fish was monitored daily for a year. From birth onwards, number of embryos derived from heterozygotes inter-crosses were counted and each dead embryos/larvae were collected and soaked immediately in DNA extraction buffer for subsequent genotyping. On 60 dpf, all fish survived were genotyped by fin-clipping, grouped into WT, *ikk2* ^+/−^ and *ikk2* ^−/−^ categories and individual death was recorded daily thereafter. Number of fish were re-counted every 5 days and any fish missing was censored (marked as a tick in Fig. 4A). Kaplan-Meier survival curve was plotted from data pooled from three independent repeats using Prism Graphpad and *p* values were computed using the log-rank test. For measurement of standard length, the linear distance between the snout and the caudal peduncle was taken to the nearest millimeter using a plastic ruler. All data are expressed as mean ± SEM. Unless otherwise stated, statistical differences were assessed by either paired or unpaired Student’s t-test using Prism Graphpad 4.0.

### Transcriptome analysis by RNAseq and bioinformatics

150 embryos for each sample were collected at 2 and 4 hpf (wild type control and MZ*ikk2* ^−/−^ for both stages, correspondingly) and RNA preparation was done as published before^58^. mRNA quantification data was obtained using a standard pipeline based on TopHat-cufflinks^59^ with the *Danio rerio* gene annotation file (assembly GRCz10) from Ensembl database. For differential expression analysis, gene-level FPKM values were converted into log ratios (KO/WT, base 2) for 2 h and 4 h data and the threshold for differential expression was determined to meet 1% FDR by a mixture model-based approach called EBprot^60^. The transcripts whose largest FPKM values was below 25 percentile of all FPKM values across the sample were considered unreliable for quantification and removed from subsequent analysis. For the genes that were quantified in either KO or WT but not both, a gene was considered differentially expressed if the quantified FPKM value^61^ was above the 25 percentile of the whole transcriptome in the given sample. Test of functional enrichment (Gene Ontology) was performed using an in-house program that computes the significance of enrichment by hypergeometric test, using the GO annotation of genes from ZFIN database (http://zfin.org). The list of NFkB target genes for zebrafish was identified by first performing gene orthology mapping to human genes (ZFIN database) and matching them to a curated list of known NFkB target genes (http://www.bu.edu/nf-kb/gene-resources/target-genes). The RNAseq data were deposited online (GEO submission no. GSE90971).

## Electronic supplementary material

Figures S1–6 with legends and Table S1
