# Supplementary material for: Ikk2 regulates cytokinesis during vertebrate development
Source: Sci Rep. 2017 Aug 14;7:8094. doi: 10.1038/s41598-017-06904-7 (PMC5556003; doi:10.1038/s41598-017-06904-7)
Supplement: Supplementary file 1 — Figures S1–6 with legends and Table S1 [file 41598_2017_6904_MOESM1_ESM.pdf]

Ikk2 regulates cytokinesis during vertebrate development

Hongyuan Shen[1], Eun Myoung Shin[1,5], Serene Lee[2], Sinnakaruppan Mathavan[2], Hiromi Koh[3], Motomi Osato[5], Hyungwon Choi[1,3], Vinay Tergaonkar[1,6,7] and Vladimir Korzh[1,4]

1- Institute of Molecular and Cell Biology, Singapore;
2- Genome Institute of Singapore, Singapore;
3- Saw Swee Hock School of Public Health, National University of Singapore
4- International Institute of Molecular and Cell Biology, Warsaw, Poland
5- Cancer Science Institute, NUS, Singapore
6- Department of Biochemistry, NUS, Singapore
7- Center for Cancer Biology, UniSA, Adelaide, Australia

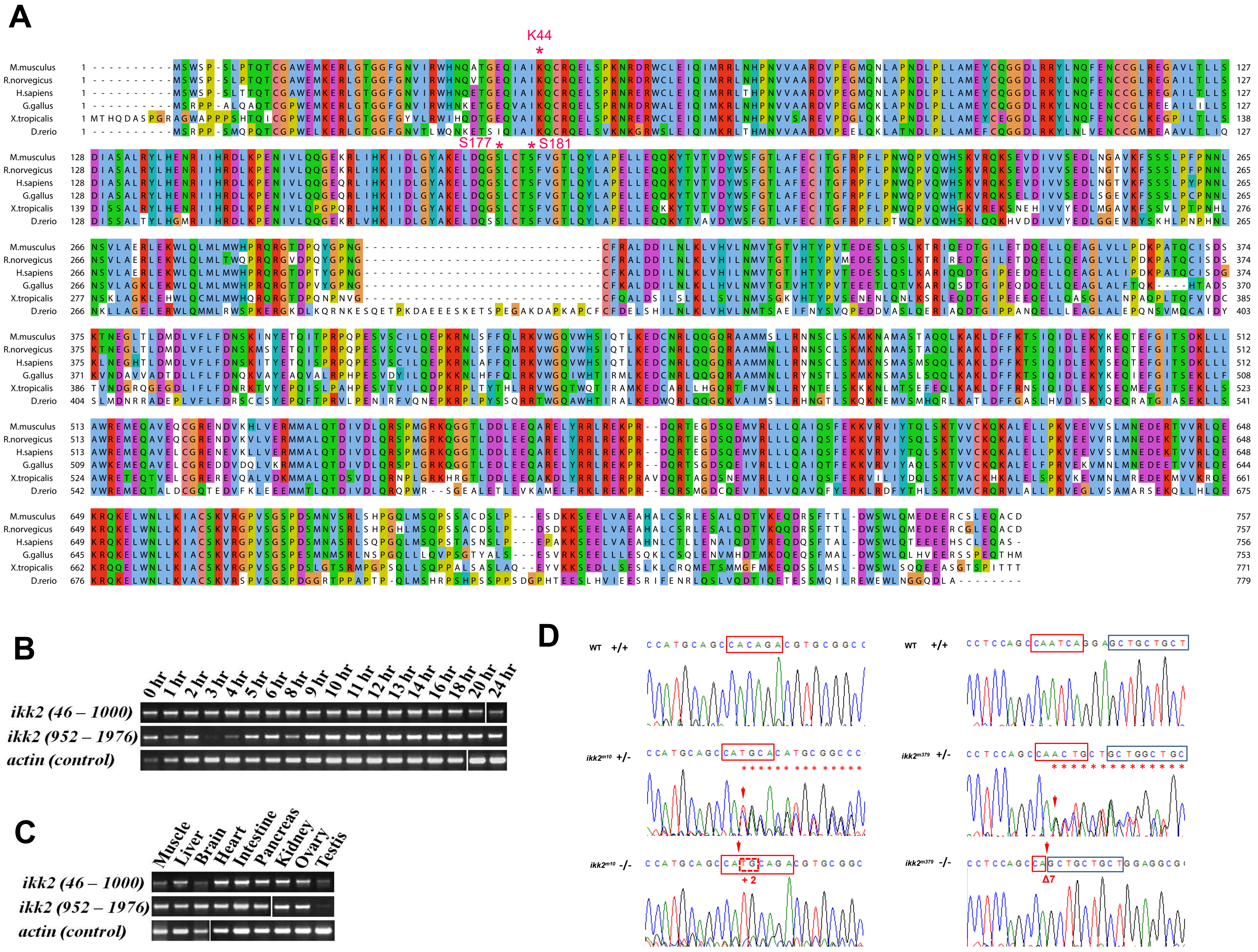

Suppl Figure 1.

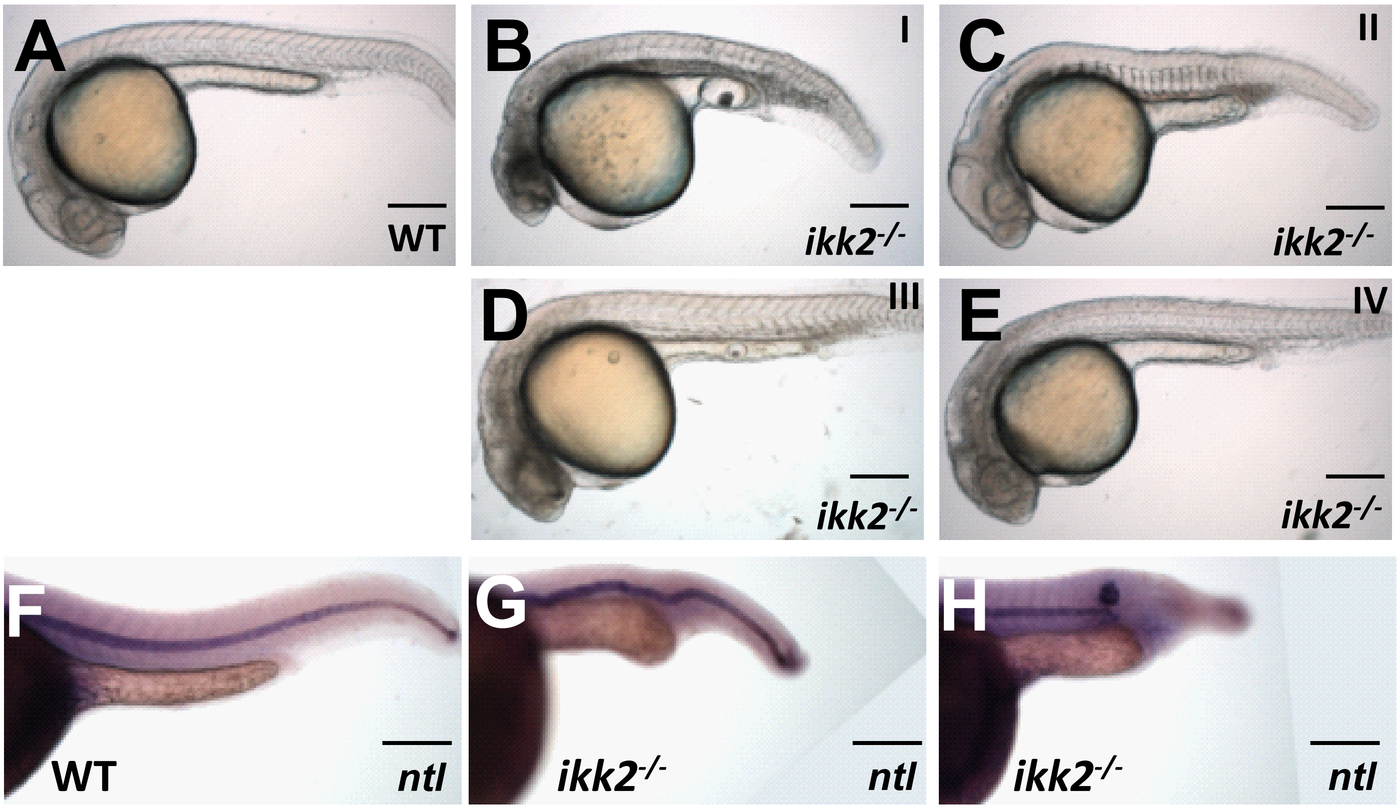

Suppl Figure 2.

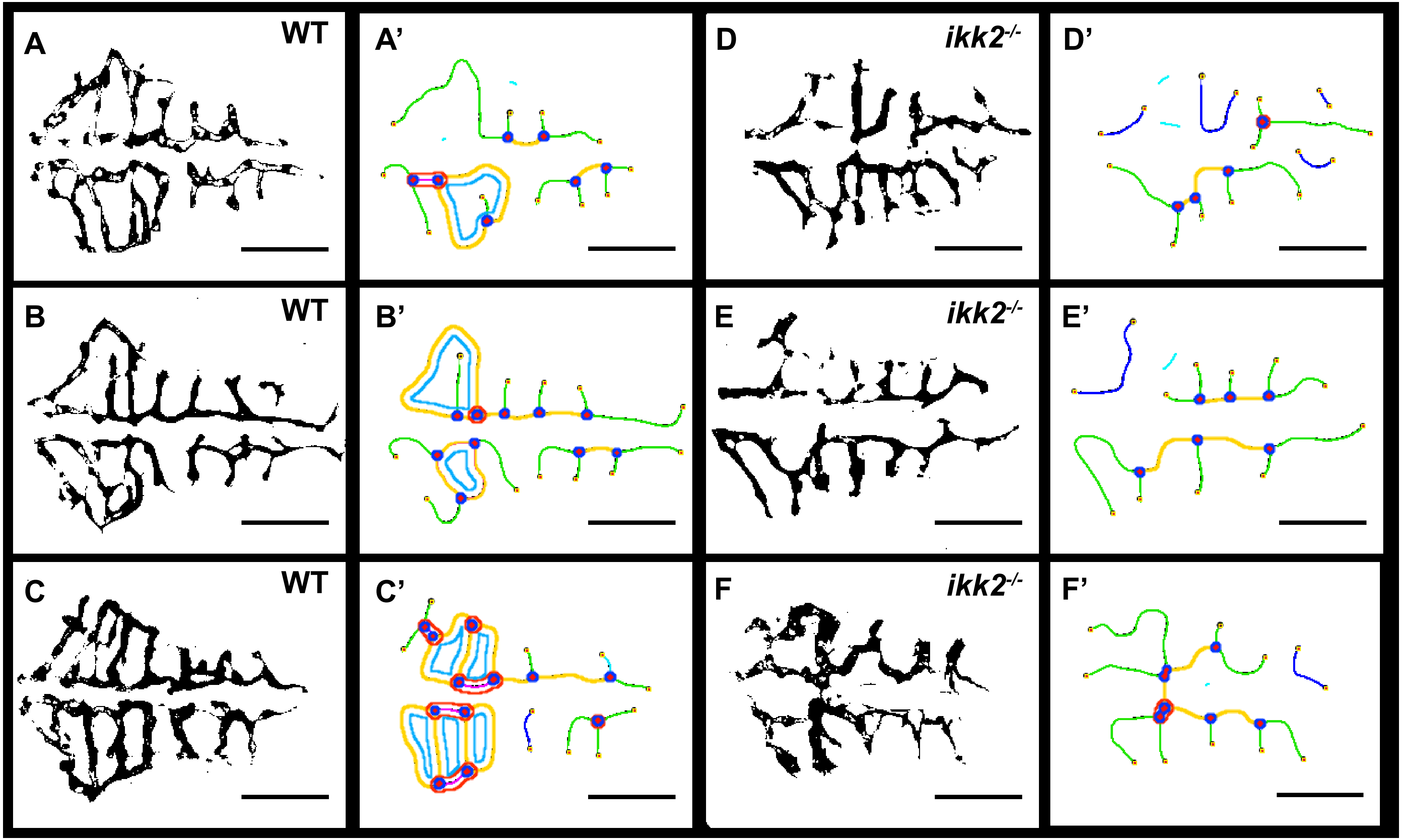

Suppl Figure 3.

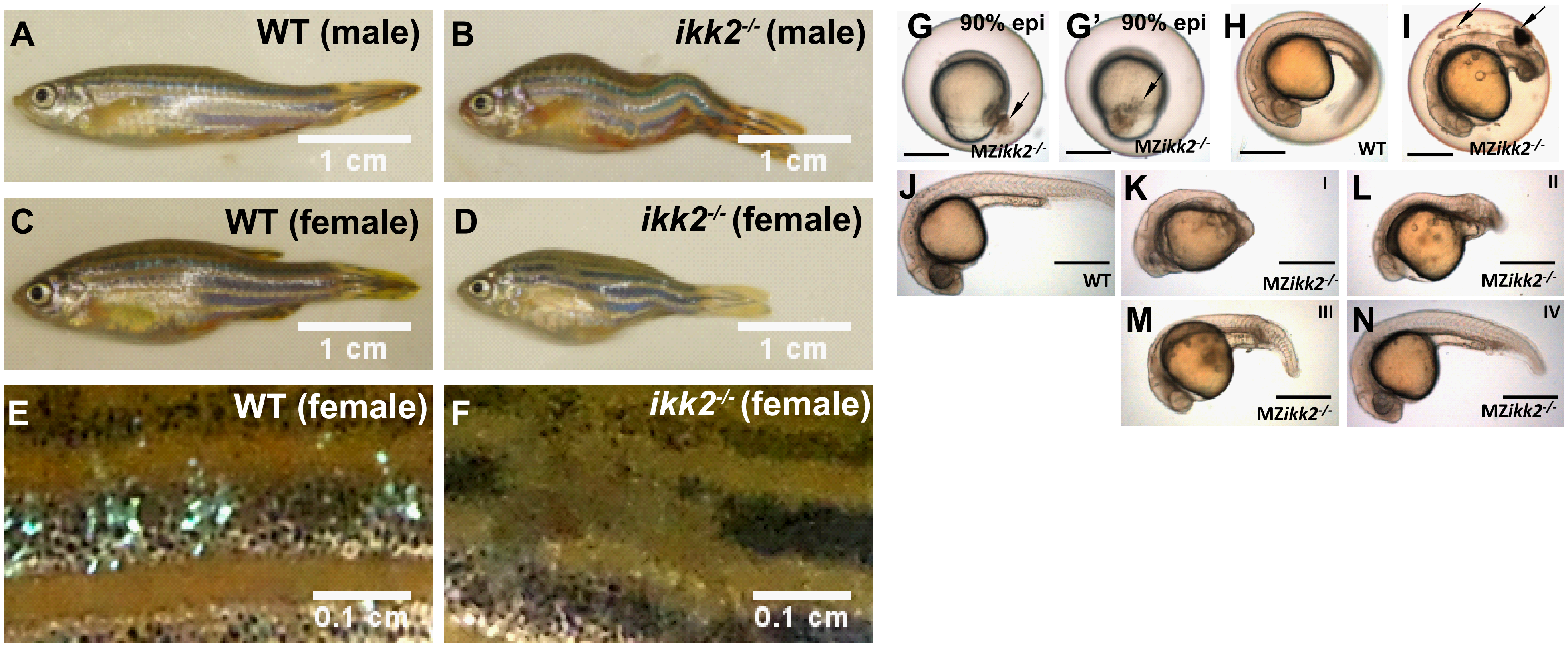

Suppl Figure 4.

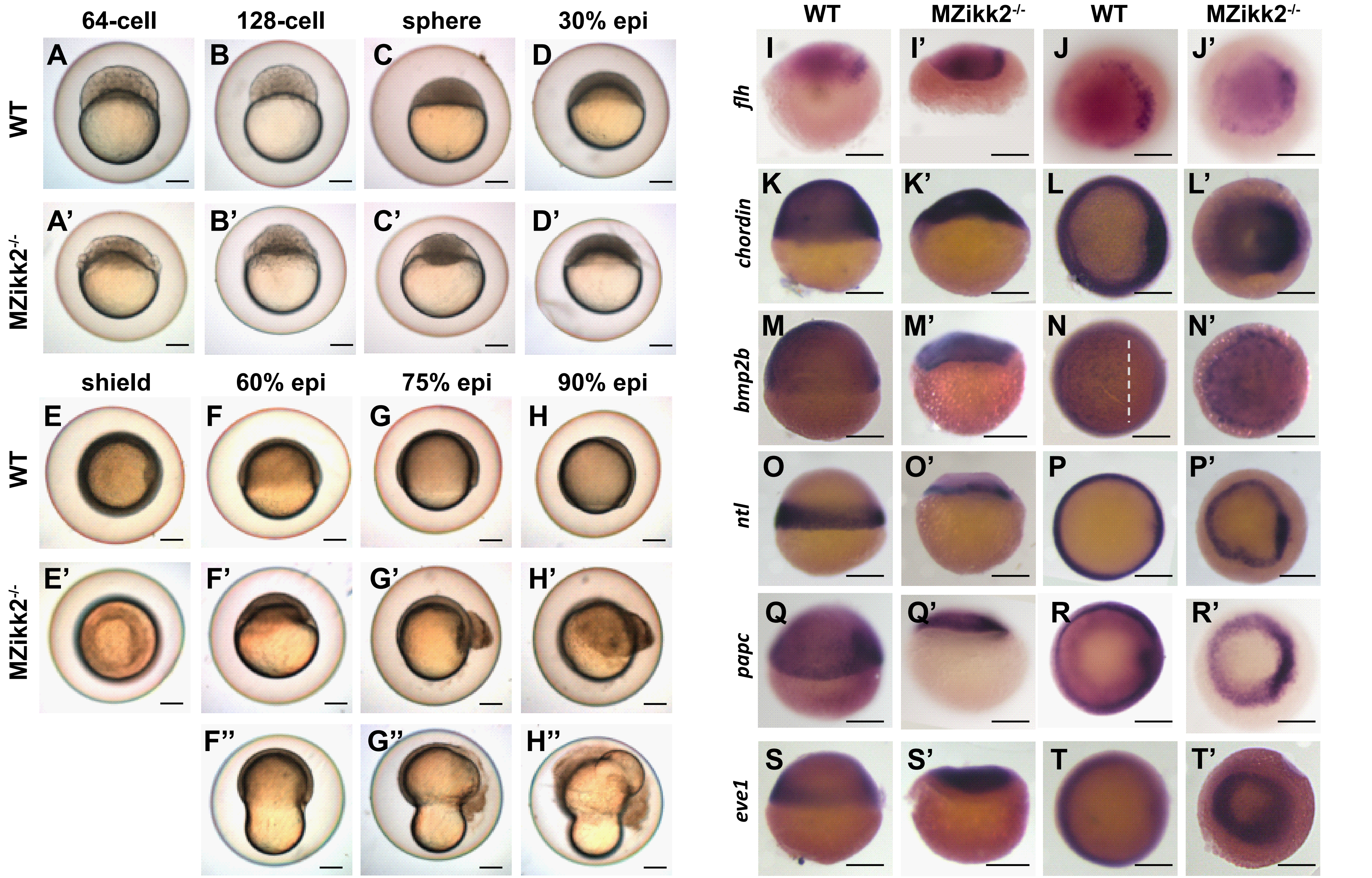

Suppl Figure 5.

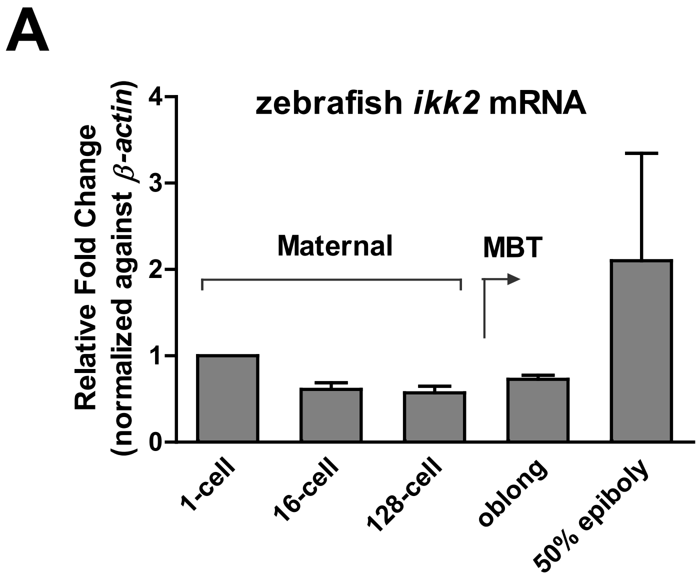

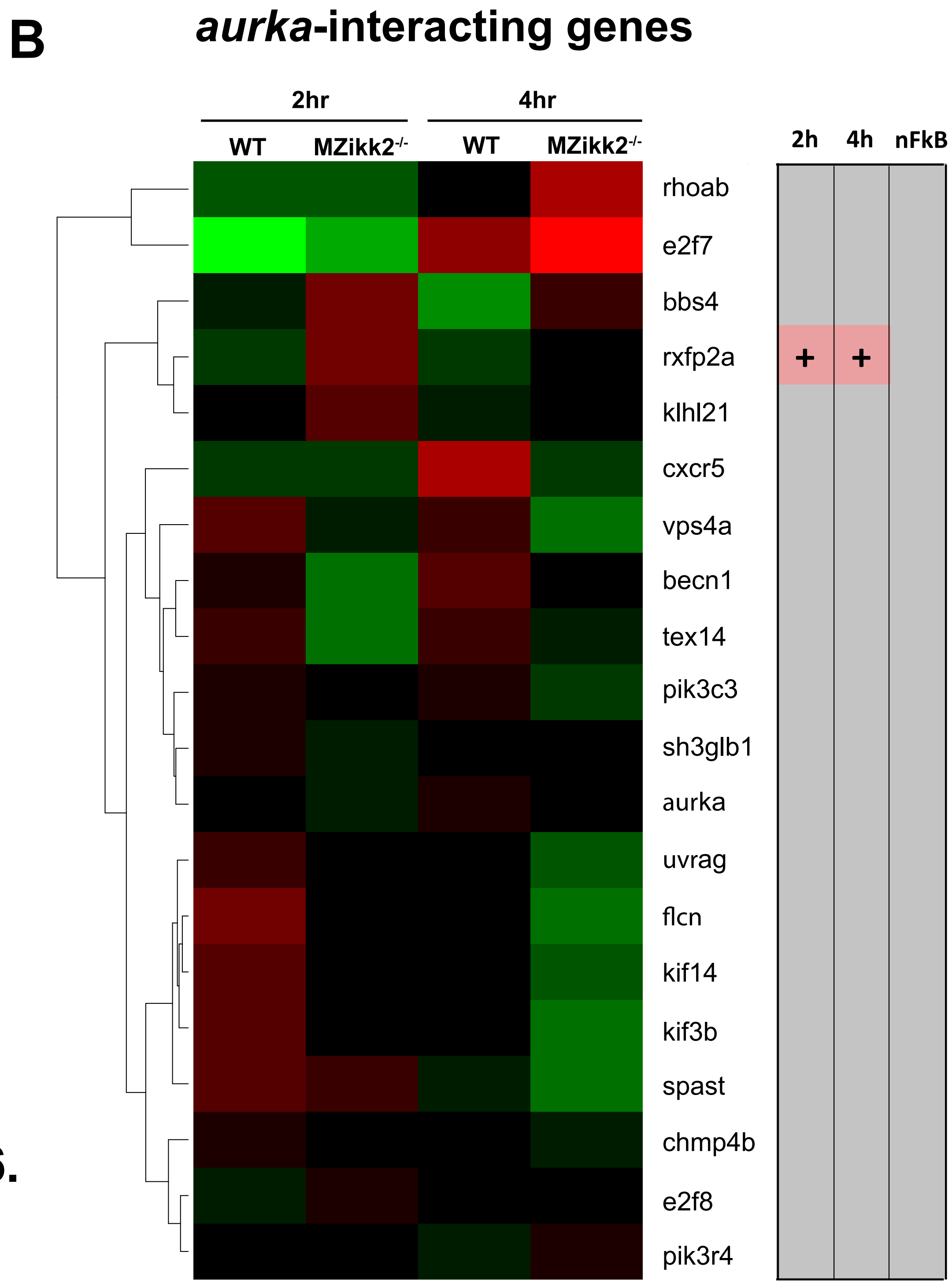

Suppl Figure 6.

Table S1

Developmental events affected by loss-of-function of Ikk2

Defect in\LOF	*MZikk2-/-*	*Zikk2-/-*	Anti-ikk2 MO
Cytokinesis	√		
YSL	√		
Gastrulation	√		√
Notochord\axis formation	√	√	√
Angiogenesis		√	
Survival as embryos	√		√
Survival as adults		√	

Supplementary Figures Legends.

Figure S1. Structure, expression and mutations of Ikk2. (A) Multiple sequence alignment between putative zebrafish Ikk2 protein and proteins from other species including human, mouse, rat, chicken and frog. Note that K44 of the catalytic active site and the two serine residues (S177/S181) of the activation loop (marked in red) are conserved between zebrafish and other species. Alignment is coloured using ClustalW alignment scheme. (B, C) Semi-quantitative RT-PCR analysis of ikk2 expression during embryogenesis and in adult organs. Two different primer pairs amplifying *ikk2* (n.t. 46-1000 and n.t. 952-1976) cDNA were used independently. *actin* was used as a loading control. (D) Example of sequence chromatograph of DNA regions containing mutations in the two ZFN-mediated zebrafish *ikk2* mutant alleles. The $ikk2^{m10}$ allele carries a two-nucleotide insertion, whereas the $ikk2^{m379}$ allele carries a 7-nucleotide deletion. All fish were genotyped by sequencing.

Figure S2. The hindbrain angiogenic vasculature of wild-type and zygotic $ikk2^{-/-}$ embryos (obtained from heterozygotic parents) at 48 hpf. (A-F), ImageJ processed binary image of the confocal projection view of the hindbrain CtA vessels. I'-G', corresponding output image from ImageJ Angiogenic Analyser Macro with corresponding vessel junctions, branches, master segments, isolated segments marked in different colours. Their length and numbers were analysed to calculate the angiogenic index using ImageJ Angiogenic Analyser Macro as shown in Figure 3, F-H.

Figure S3. Analysis of zygotic mutants at 24 hpf. (A-E) Bright-field lateral view of wild-type and $ikk2^{-/-}$ (zygotic) embryos obtained from heterozygotic parents with notochord defects at 24 hpf. A, wild-type embryos. B-E, $ikk2^{-/-}$ embryos classified into four categories

based on severity of the notochord defects. Class I and II show notochord defects and U-shaped somites. Class III and IV show mild notochord defects and increased turbidity of the brain suggestive of apoptosis. (F-H) Lateral view of embryos stained by *in situ* hybridization using *ntl* probe. *ikk2*$^{-/-}$ embryos have either crooked notochord or its truncation. Scale bar: 250 μm.

Figure S4. Morphological analysis of adult heterozygotic and embryonic homozygotic Ikk2 mutants obtained from heterozygotic parents. (A-D) Adult *ikk2*$^{-/-}$ fish with skeletal defects. Adult *ikk2*$^{-/-}$ fish are short with curved skeleton (B, D) as compared to wild types (A, C). (E, F) Adult *ikk2*$^{-/-}$ fish are prone to skin lesion accompanied by loss of scales and hypopigmentation. Scale bar in A-D: 1cm; scale bar in E,F: 0.1 cm. (G-N) In *MZikk2*$^{-/-}$ escapers cells detach in embryonic shield (arrow) at late gastrula. (G, G'), later stage embryos contain increased cell debris under the chorion (I, arrowed) and show defects in posterior body (K-N). G, lateral view. G', dorsal view. J-N, lateral view, 24 hpf. K-N, *ikk2*$^{-/-}$ embryos with defects in posterior body are classified into four categories (I to IV) based on severity of the notochord defects. This phenotype mimics that of zygotic *ikk2*$^{-/-}$ mutants (Figure S2, A-E). Scale bar in G-N: 500 μm.

Figure S5. Morphological and WISH analysis of *MZikk2*$^{-/-}$ embryos. (A-H) Bright field image of wild-type and *MZikk2*$^{-/-}$ embryos obtained from homozygotic parents at blastula and gastrula stages. *MZikk2*$^{-/-}$ embryos show disorganized blastoderms (A'-C') unlike compact blastoderm of wild-type embryos (A-C). Epiboly is incomplete (D'-H') and cells detach from yolk. F''-H'', due to delayed epiboly constriction starts before epiboly completion resulting in peanut-shaped embryos. All embryos shown in lateral view. (I-T) Wild-type and *MZikk2*$^{-/-}$ embryos stained by WISH at shield stage. Embryos stained with dorsal markers (*flh*, *chordin*

in I-L), ventral markers (*bmp2b* in M-N) , and mesendoderm markers (*ntl*, *papc*, and *eve1* in O-P). *MZikk2*$^{-/-}$ embryos show signs of correct dorsal-ventral patterning and mesendoderm differentiation. I, K, M, O, Q, S: lateral view with dorsal side to the right; J,L,N,P,R,T: animal pole view with dorsal side to the right. Scale bar in all panels: 200 µm.

Figure S6. (A) Real-time PCR (qPCR) quantification of *ikk2* expression during early embryonic stages. qPCR results were normalized against actin (*actb*), and expressed as relative fold changes as compared to 1- cell stage using the ΔΔCt methods. Error bars represent mean ± SEM. (B) Heat-map representing RNAseq results of *aurka* and its interacting partners (green – positively-regulated gene, red – negatively-regulated gene).
